# PREDICTORS OF PHYSICAL FUNCTION DECLINE AMONG OLDER ADULTS RECEIVING HOME CARE NURSING: PROSPECTIVE COHORT STUDY

**DOI:** 10.1093/geroni/igad104.3775

**Published:** 2023-12-21

**Authors:** Taisuke Yasaka, Ayumi Igarashi, Chie Fukui, Asa Inagaki, Yuka Sumikawa, Manami Takaoka, Sameh Eltaybani, Noriko Yamamoto-Mitani

**Affiliations:** The University of Tokyo, Tokyo, Tokyo, Japan; The University of Tokyo, Tokyo, Tokyo, Japan; YuPia Inc., Nagoya, Aichi, Japan; The University of Tokyo, Tokyo, Tokyo, Japan; The University of Tokyo, Tokyo, Tokyo, Japan; The University of Tokyo, Tokyo, Tokyo, Japan; The University of Tokyo, Tokyo, Tokyo, Japan; The University of Tokyo, Tokyo, Tokyo, Japan

## Abstract

Older adults receiving home care nursing have certain health conditions and face continuous risk of declining physical functions. Identifying those at risk of functional decline based on these factors is essential for developing appropriate strategies for maintaining their conditions. This prospective cohort study in home care nursing agencies across Japan examined ADL decline incidence and its factors among clients aged ≥75. Data were collected from nurses in charge of clients using online questionnaires. Functional status was evaluated using the ADL Hierarchy (ADLH) score, ranging from 0 (independent) to 6 (dependent). A binary logistic regression examined the relationships between clients’ characteristics at baseline and ADLH score deterioration after 6 months, controlling for ADLH score at the baseline. Of the 715 clients analyzed, 31% had deteriorated ADLH scores between baseline and 6 months later. Older age (Adjusted odds ratio 1.04 [95% confidence interval 1.01–1.07]), having neurology diseases (2.23 [1.06–4.69]), use of home visiting physicians (1.54 [1.04–2.27]), falls with/without trauma in the previous 6 months (2.77 [1.41–5.44], 1.90 [1.11–3.25]), higher ADLH score at baseline (1.35 [1.18–1.56]), higher severity of dementia (1.23 [1.02–1.47]), occurring dyspnea in the previous 30 days (1.69 [1.05–2.71]), and having BMI lower than 18.5 (between 25 and 30 as a reference) (2.58 [1.12–5.94]) were associated with ADLH score deteriorations. The results suggest that inhibiting functional decline among older adults receiving home care nursing might be possible by optimizing the management of modifiable predictors such as frequent falls, dyspnea, and weight loss.

